# Comparison of Approaches to Determining the Coefficient of Friction in Stretch-Forming Conditions

**DOI:** 10.3390/ma18194534

**Published:** 2025-09-29

**Authors:** Tomasz Trzepieciński, Krzysztof Szwajka, Valmir Dias Luiz, Joanna Zielińska-Szwajka, Marek Szewczyk

**Affiliations:** 1 Department of Manufacturing Processes and Production Engineering, Faculty of Mechanical Engineering and Aeronautics, Rzeszów University of Technology, al. Powstańców Warszawy 8, 35-959 Rzeszów, Poland; 2 Department of Integrated Design and Tribology Systems, Faculty of Mechanics and Technology, Rzeszów University of Technology, ul. Kwiatkowskiego 4, 37-450 Stalowa Wola, Poland; kszwajka@prz.edu.pl (K.S.); m.szewczyk@prz.edu.pl (M.S.); 3 Department of Metallurgy and Chemistry, Centro Federal de Educação Tecnológica de Minas Gerais, Timóteo 35180-520, Brazil; valmir@cefetmg.br; 4 Department of Component Manufacturing and Production Organization, Faculty of Mechanics and Technology, Rzeszów University of Technology, ul. Kwiatkowskiego 4, 37-450 Stalowa Wola, Poland; j.zielinska@prz.edu.pl

**Keywords:** electron beam melting, friction, ion implantation, sheet metal forming, steel sheets, stretch forming

## Abstract

Control of the friction process in stretch-forming conditions, when creating sheet metal, is essential for obtaining components of the quality required. This paper presents an approach to modelling the friction phenomenon at the rounded edges of stamping dies. The aim of the study is to compare the coefficient of friction (CoF) determined from numerous analytical models available in the literature. Experimental studies were conducted using self-developed bending under tension friction testing apparatus. The test material was low-carbon DC01 steel sheeting. Tests were conducted under lubricated conditions, using industrial oil intended for deep drawing operations. The surfaces of countersamples made of 145Cr6 substrate were modified using the ion implantation of Pb (IOPb) and electron beam melting processes. Variation in the CoF in BUT tests was related to continuous deformation-induced changes in surface topography and changes in the mechanical properties of sheet metal due to the work-hardening phenomenon. Under friction testing with a stationary countersample, the largest increase in average roughness (by 19%) was found for the DC01/IOPb friction pair. The friction process caused a significant decrease in kurtosis values. The results show that the difference between the highest and lowest CoF values, determined for the analytical models considered, was approximately 40%.

## 1. Introduction

The deep drawing of sheet metals is the most effective method for forming components in the automotive industry. The selection of forming technology is crucial for ensuring the stability and efficiency of the deep drawing process [1,2]. The assessment of sheet metal’s susceptibility to deep drawing is performed using standardised uniaxial or biaxial tensile tests and formability tests. In addition to the mechanical properties of the workpiece, its behaviour during processing is also influenced by friction conditions [3,4]. Friction is a complex phenomenon occurring at the interface between the formed sheet metal and the tool surface. Draw forming takes place under stretch-forming conditions [5]. This causes friction conditions to constantly change due to deformation-induced changes in the sheet metal topography and changes in the mechanical properties of the sheet material related to strain hardening [6,7]. Under these conditions, controlling the sheet metal forming process is extremely difficult.

Controlling friction conditions in deep drawing processes involves the selection of the appropriate tool material and, possibly, the application of wear-resistant protective coatings [8]. Such a formation of tool surface is aimed at increasing its hardness, thus reducing its wear [9,10]. Due to high contact pressures, the flattening of surface asperities and adhesion are typical phenomena accompanying the sheet metal forming process [11,12]. Forming force parameters are also sensitive to friction. Process variables such as sliding velocity, processing temperature, amount and distribution of contact pressure, surface roughness of the friction pair components, type of lubricant, topography of the tool surface, and tribological properties of the tool surface, can significantly affect friction conditions in sheet metal forming processes [13,14]. Reducing friction is essential to improving the stability of the forming process and lubrication is the most effective way of reducing friction [15,16].

An effective tool for optimising the process of designing sheet metal forming technologies is numerical simulation, mainly using the finite element (FE) method [17,18]. Knowledge of friction conditions is essential for correctly predicting the stress state in the material and preventing the unfavourable phenomena of sheet metal wrinkling and premature cracking. The integration of experimentally derived friction coefficients (CoFs) from BUT tests into FE-based numerical models has been shown to significantly improve the predictive capability of simulations, particularly in relation to formability [19] and springback [20]. The tribological properties of sheets are assessed in simple friction tests, which allow for the determination of an averaged coefficient of friction (CoF) for the considered friction pair. The strip drawing test is most commonly used for this purpose, but it does not accurately reflect the friction conditions at a rounded tool edge due to the lack of correlation with stretch-forming conditions. However, studies are available on the effect of deformation-induced surface/interface roughening of a sheet on the frictional properties [21,22].

The evaluation of friction conditions on the edges of stamping tools is performed using bending under tension (BUT) friction tests. In these tests the sheet metal undergoes elongation as it passes over the rounded tool edge. The BUT test replicates the contact conditions and deformation behaviour commonly encountered in forming operations, where sheet metal is subjected to a combination of bending over a die radius and axial tension. Previous studies employing the BUT test have demonstrated its effectiveness in capturing the influence of material properties, sliding speeds, surface topography, and lubrication on the frictional behaviour of workpiece/tool friction pairs. Wilson et al. [23] developed a BUT apparatus where the contact angle and the relative sliding velocity between the tool and strip sample can be varied. Swift [24] separated the effects of friction and bending in the BUT test by developing an apparatus in which the pin (countersample) rotates or remains stationary. Saha and Wilson [25] calculated the CoF in BUT tests from the equation proposed by Wilson et al. [23], with an allowance for bending according to the approach developed by Swift [24]. This approach allowed the determination of the effects of strain rate, strip strain, and sliding speed on the CoF. In the approach proposed by Saha and Wilson [25], determining the CoF in BUT tests based on force parameters requires conducting two tests, involving a rotating and stationary pin (countersample). Andreasen et al. [26] and Sniekers and Smits [27] used a torque sensor on the BUT test pin to eliminate the second step of the free pin test.

Much research has also been devoted to the use of the BUT test and its variants to modify material properties and analyse formability and springback phenomena. Shih [28] used BUT tests to investigate the bendability of advanced high-strength steels (AHSS). He proposed the use of limiting bending strains as the die thinning criteria. Sharma et al. [29] conducted a continuous BUT test to assess the elongation-to-failure performance of AHSS transformation-induced plasticity (TRIP) steels. It was found that avoidance of the localisation of plastic strain during testing accounted for increased sample elongation. Hudgins et al. [30] investigated the stress state experienced by the die radius when testing some grades of AHSS. The tests allowed for the assessment of failure mode for various pin radius/sheet thickness ratios. Wagner et al. [31] used BUT tests to analyse the springback phenomenon in AHSS. They found that, despite the lack of change in Young’s modulus, the movement of dislocations can cause nonlinearity of the unloading curve, which is taken into account in SMF simulations. Similarly, Zheng et al. [32] used BUT tests in a numerical study of the effect of a hardening model on the springback of dual-phase DP980 steel sheets. They suggested that the Yoshida–Uemori model can achieve better prediction accuracy for springback compared to the Swift material model. Matukhno et al. [33] used continuous BUT tests to evaluate the strength and elongation-to-fracture of AZ31 sheets. Experimental results revealed that the strength of the test material can be increased by over 30%. Ha et al. [34] investigated the combination of pre-forming heat treatment and continuous BUT tests to evaluate the formability of EN AW-5754-H32 alloy sheets. Mayer et al. [35] optimised the ductility and strength of EN AW-5182-O alloy sheets through a combination of annealing processing and cyclic bending under tension. It was found that a combination of heat treatment and continuous BUT tests can increase strength by over 75%. Tamimi et al. [36] modified the microstructure of EN AW-7075 alloy sheets by repetitive BUT tests. The results revealed that the formability of test sheets can be increased by 220% compared to their standard formability.

Previous analytical solutions were based on the assumption of uniform pressure at the contact interface. Folle and Schaeffer [37] proposed an equation for determining CoF based on the pin torque and vertical force. As noted by Nielsen et al. [38], torque and horizontal force equilibriums cannot coexist. Therefore, they proposed an alternative determination of the friction coefficient based on vertical force, horizontal force, and torque equilibrium. Sniekers and Smits [27] proposed an FE-based model to determine the pressure distribution in the BUT testing. It was found that the pressure distribution is inhomogeneous with two peaks near the outlet and inlet of the contact interface. This conclusion was later confirmed in the FE-based numerical works of Kim et al. [39], Trzepieciński and Lemu [40,41], Pereira et al. [42], and Ceron and Bay [43], among others.

In the literature, several equations are currently used to determine friction coefficients in the BUT test. However, experimental studies demonstrating quantitative differences in friction coefficients determined by different methods are lacking. This paper presents the results of laboratory-scale friction studies using a self-developed BUT apparatus. The experimental test system is described, along with the consideration of multiple analytical approaches to determine the coefficient of friction. Various methods of modifying the surface of countersamples resulting from ion implantation and electron beam melting are also considered. Overall, this research contributes to the development of the BUT test as a reliable and practical method for characterising friction in sheet metal forming. The presented methodologies and results contribute to the improvement of material models and support the development of more reliable and efficient methods for determining the CoF in industrial applications.

## 2. Materials and Methods

### 2.1. Test Material

The material used for friction testing via the BUT tests was DC01 steel sheet with a thickness of 0.8 mm. This is a low-carbon steel with a carbon content of no more than 0.12%. It also contains small amounts of manganese, phosphorus, sulphur, and other alloying elements in minimal amounts. Basic mechanical properties determined in a uniaxial tensile test, in accordance with the standard [44], resulting in an ultimate tensile strength of 290.2 MPa, a yield stress of 163.4 MPa, and an elongation of 37.6% (gauge length of 50 mm). Due to its low carbon content and deep drawability, DC01 steel sheet is used for deep-drawn components with complex shapes.

**Figure 1:**
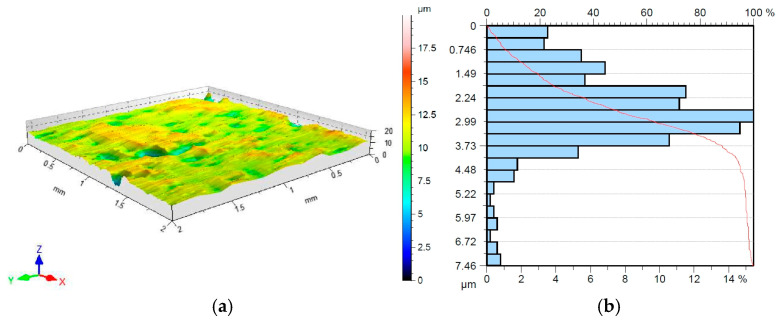
(**a**) Topography; (**b**) the bearing area curve of the DC01 steel sheet (as-received condition).

The topography of the sheet metal in its as-received condition (Figure 1), and after friction testing was examined using a Hommel-Etamic T8000RC profilometer (Jenoptik, Jena, Germany), in accordance with the standard [45]. The basic surface roughness parameters of the DC01 steel sheet in as-received condition are listed in Table 1.

### 2.2. Experimental Procedure

**Figure 2:**
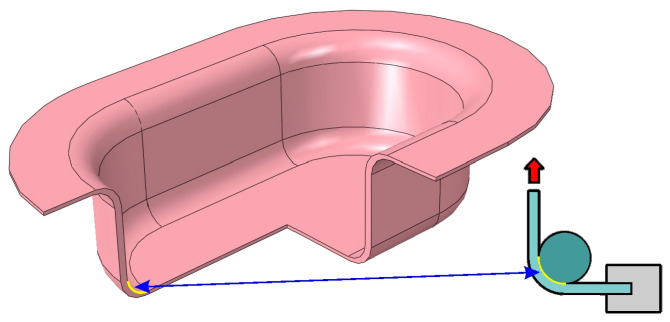
The area of application of the BUT test in deep drawing processes.

Friction tests were conducted using a self-developed tribotester, which allows for a two-step procedure to separate the bending effects in the BUT test, with a wrap angle of 90°, corresponding to typical deep drawing processes, where a punch with a rounded edge contacts the sheet metal (Figure 2). The wrap angle analysed was consistent with the research methodology of other authors, including [23,26,27,37].

**Figure 3:**
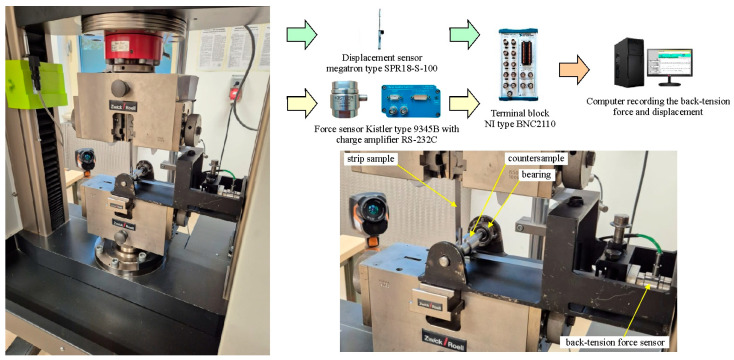
Schematic of the BUT test apparatus.

The device consisted of a housing containing a bearing-mounted shaft with a cylindrical countersample (radius of 15 mm), which corresponds to the range of radii of punch edges used when forming sheet metals [46]. A freely rotating countersample allowed for separate bending effects in the BUT test [24,25]. A horizontally positioned holder equipped with a type 9345B Kistler piezoelectric force sensor (Kistler, Winterthur, Switzerland) was used to secure one end of a 400 mm long and 18 mm wide strip sample. The other end of the strip sample was mounted in the upper grip of a Zwick Z100 testing machine (Zwick/Roell, Ulm, Germany). The device housing was mounted in the lower grip of the testing machine (Figure 3).

**Figure 4:**
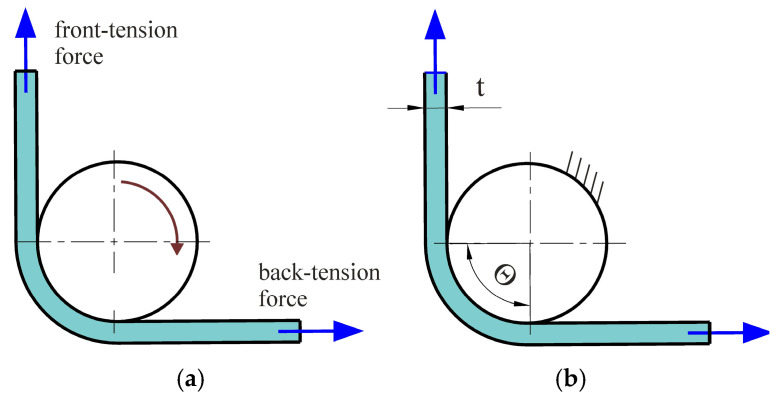
Schematic of two-stage BUT test under (**a**) a freely rotating and (**b**) stationary countersample.

In the first stage, the friction test was performed with a freely rotating countersample. In the second stage, the countersample was prevented from rotating freely. During both stages, front-tension and back-tension forces (Figure 4) were recorded. The front-tension force was recorded by the measuring system of the tensile testing machine. Back-tension force was recorded using a type 9345B Kistler force sensor and a LabView environment (National Instruments Corporation, Austin, TX, USA). The values of both forces were then correlated using a Megatron Series SPR18 displacement sensor (Megatron Elektronik GmbH & Co. KG, Putzbrunn, Germany). The coefficient of friction was determined based on the various approaches described in Section 2.4.

**Figure 5:**
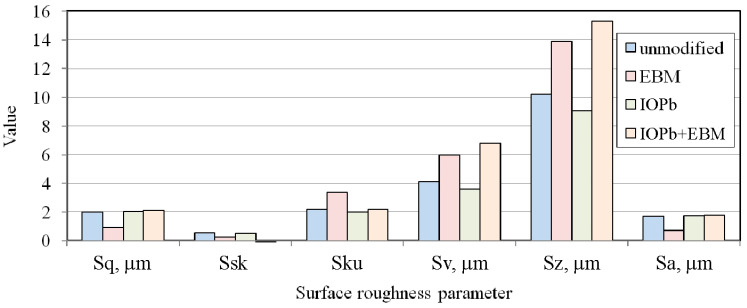
Surface roughness parameters of the countersample.

**Figure 6:**
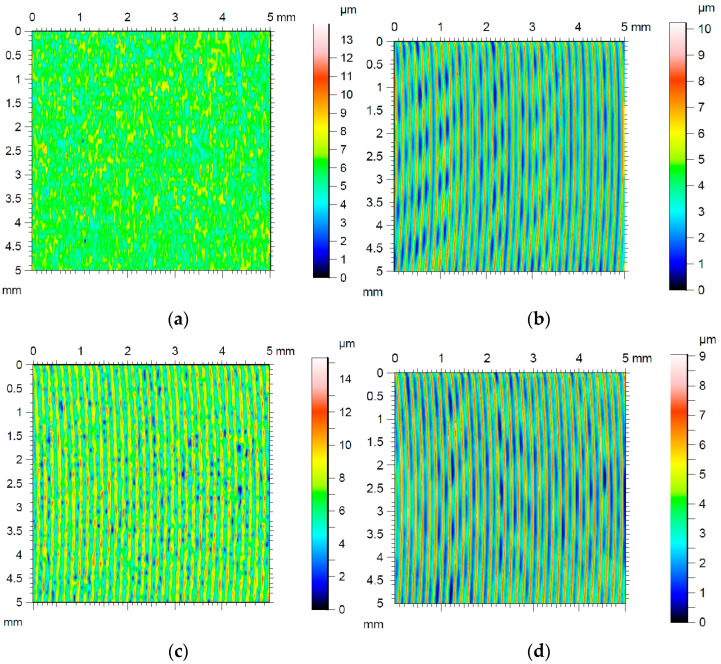
Surface topography of countersample: (**a**) EBM, (**b**) unmodified, (**c**) IOPb+EBM, and (**d**) IOPb.

Cylindrical countersamples were prepared with different working surfaces. The base (unmodified) countersample was made of 145Cr6 tool steel (hardness 250HV). The countersamples’ surfaces were modified by the ion implantation (IO) of Pb (IOPb), electron beam melting (EBM), and combined ion implantation of Pb and EBM (IOb+EBM). The preparation procedure of countersamples, IO, and EBM process parameters were presented in a previous study [6]. The lowest average roughness was characteristic for countersamples after the EBM process (Figure 5). The remaining countersamples were characterised by more than twice the average roughness (Sa = 1.7–1.81 μm). Figure 6 presents the topography of the countersample.

Tests were conducted using S 100+ oil (Naftochem sp. z o.o., Kraków, Poland). This oil is specially formulated for the industrial deep drawing operations of drawpieces with complex geometries in the sheet metal forming industry. Before applying the oil, the sheets and the countersamples were cleaned.

### 2.3. Evaluation of Contact Pressure

The Prescale pressure measuring film (FuJiFilm Corp., Tokyo, Japan) was used to determine the contact pressure values in the BUT test. Fujifilm’s Prescale film is an innovative product for measuring pressure distribution and its range. It turns red depending on the pressure applied, enabling a visual and quantitative analysis of contact pressure distribution. The Prescale film consists of two layers: a base layer with dye microcapsules (layer A) and a base layer with an activator (layer C). When pressure is applied to the film, the microcapsules in layer A rupture, releasing the dye, which reacts with the activator in layer C. The measurement accuracy of the Prescale film is sensitive to tangential slip, so contact pressure measurements were performed during a test with a freely rotating countersample. Based on image analysis, the relationship between sample colour and contact pressure [47] was determined.

**Figure 7:**
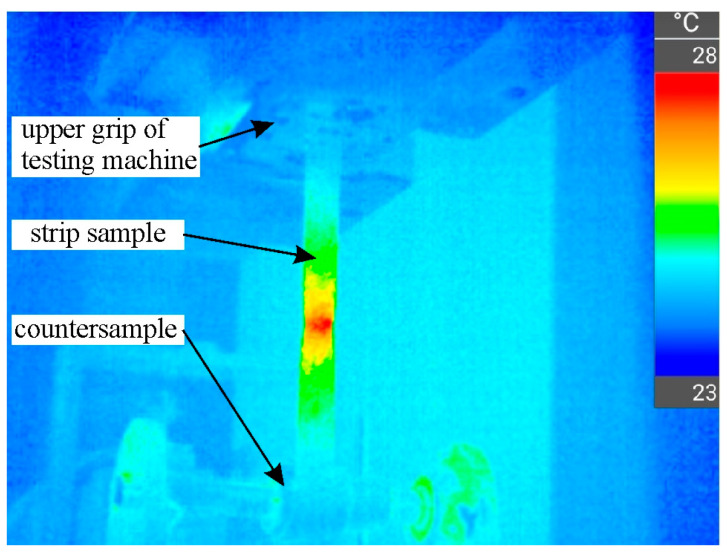
Thermograph of strip sample during BUT test at the moment of local necking.

The Prescale film is designed to measure pressures within a temperature range between 20 and 35 °C. Strain-induced internal friction of the sample did not cause significant heating of the sample surface. The sample surface temperature in contact with the countersample was approximately 24.5 °C (Figure 7). A Testo model 883 thermal imaging camera (Testo Sp. z o.o., Pruszków, Poland) was used to measure the temperature of the sample surface.

### 2.4. Determination of CoF

**Figure 8:**
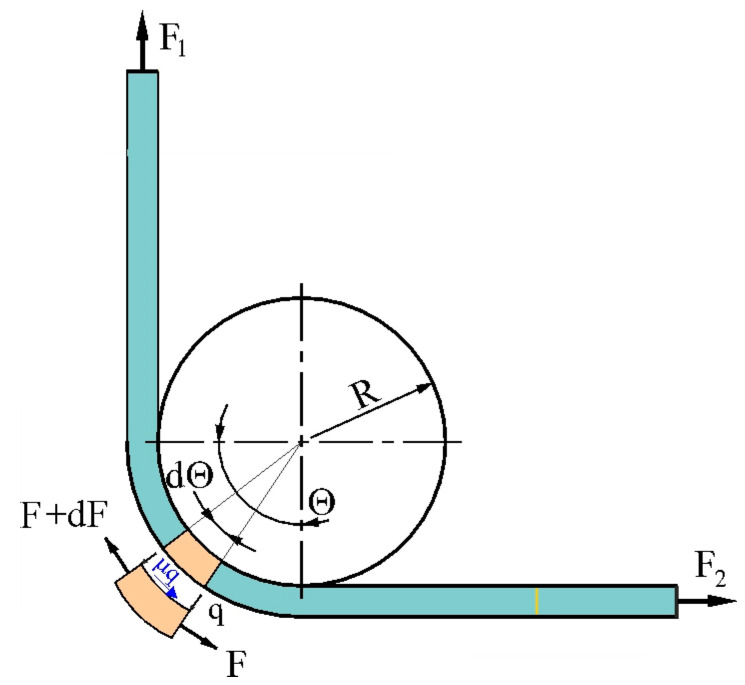
Forces acting on an elementary sample section with an angle of wrap equal to 90°.

Determining the coefficient of friction in the BUT test developed by Duncan et al. [48] involves measuring the force parameters of the friction process using a fixed countersample. Figure 8 shows the variation in the forces around the fixed countersample. The equilibrium of forces in the tangential direction gives the following [48]:

(1) $$\begin{array}{c} \mathrm{dF}=\;\mu \cdot q\cdot R\cdot w\cdot d\\ \frac{dF}{F}=\mu d\theta \end{array}$$

where μ is the CoF, R is the radius of countersample, q is contact pressure, and w is the strip width.

Integrating Equation (1), assuming μ = constant gives

(2) $$\frac{F_{1}}{F_{2}}=exp(\mu ∙\theta )$$

And, after transformations,

(3) $$\mu =\frac{1}{\theta }\ln\left(\frac{F_{1}}{F_{2}}\right)$$

Taking into account an angle of wrap equal to 90°, Equation (3) gives

(4) $$\mu =\frac{2}{\pi }ln\left(\frac{F_{1}}{F_{2}}\right)$$

**Figure 9:**
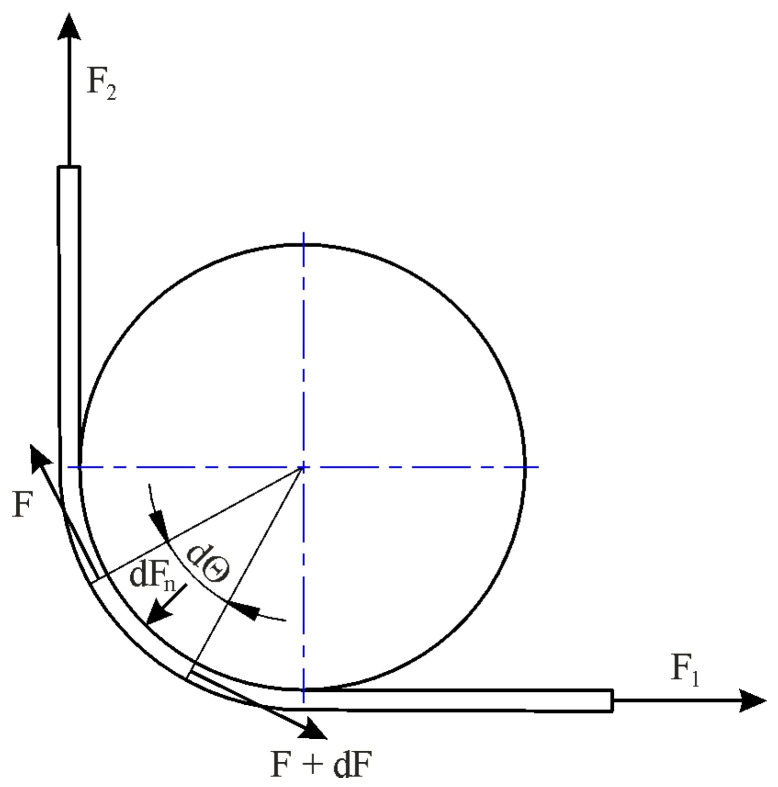
Forces acting on the countersample under stretch-forming conditions.

Fox et al. [49] proposed a modification of Equation (4). Determining the friction coefficient value in the BUT test involves measuring force parameters in two variants: with a fixed countersample and with a free countersample. The difference in the tensile forces in both test variants allows for separating the friction force from the total stretching force of the sample and, on this basis, calculating the CoF value. Figure 9 illustrates the forces acting on a specimen during the friction test. F_l_ is the applied forward tension, F_b_ is the front-tension force due to bending/unbending, and F_2_ is the resulting back-tension force.

From a force balance, the following equilibrium equations can be developed [49]:

(5) $$\mathrm{dF}\;=\;\mu \;dF_{n},\;dF_{n}\;=\;F\;d\Theta$$

Substitution of dF_n_ gives

(6) $$\int_{x}^{y}\frac{dF}{F}=\int_{0}^{\theta }\mu \;d\theta$$

where the integration range of the force parameters is between x = F_2_ and y = F_1_ − F_b_. Calculation of definite integrals in Equation (6) yields

(7) $$\mu =\frac{1}{\theta }ln\left(\frac{F_{1}-F_{b}}{F_{2}}\right)$$

Taking into account the angle of wrap equal to 90° gives

(8) $$\mu =\frac{2}{\pi }ln\left(\frac{F_{1}-F_{b}}{F_{2}}\right)$$

The test performed with free countersample simulates the frictionless condition (μ = 0) and then Equation (8) reduces to F_2_ + F_b_ = F_1_ [49].

In Equations (4) and (8), the friction coefficient does not depend on the geometry (dimensions) of the countersamples and the thickness of the strip sample. Research into taking these parameters into account was first undertaken by Han [50], who proposed an equation for determining CoF in the special case of a 90° bend:

(9) $$\mu =\frac{2}{\pi }\left(\frac{2R+t}{2R}\right)ln\left(\frac{F_{1}-F_{b}}{F_{2}}\right)$$

where t is the sheet thickness and R is the radius of the cylindrical countersample.

**Figure 10:**
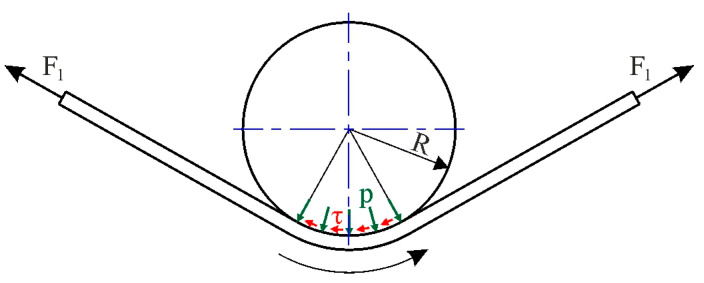
Loads on the strip in the BUT test under the assumption of uniformly distributed friction stress and contact pressure.

Wilson et al. [23] first developed an equation for determining CoF that did not take into account logarithmic relationships. They assumed a uniform pressure and friction stress distribution in the contact zone (Figure 10).

Vertical force distribution gives the following [38]:

(10) $$\left(F_{1}+F_{2}\right)sin\frac{\theta }{2}=2p∙R∙w∙sin\frac{\theta }{2}$$

After simplifications we get contact pressure p from

(11) $$p=\frac{F_{1}+F_{2}}{2w∙R}$$

From moment equilibrium

(12) $$\left(F_{1}-F_{2}\right)R=\tau ∙w∙R^{2}\theta$$

the friction stress τ can be found as

(13) $$\tau =\frac{F_{1}-F_{2}}{w∙R∙\theta }$$

After taking into account the fact that $\mu =\frac{p}{\tau }$, then for a special case of a 90° bend and the friction coefficient can be determined from Equation (14):

(14) $$\mu =\frac{4}{\pi }\left(\frac{F_{1}-F_{2}}{F_{1}+F_{2}}\right)$$

Saha and Wilson [25] proposed the modification of Equation (14) by taking into account the bending and unbending effects of the strip on the front-tension force F_1_. After subtracting F_b_ from the F_1_ force, the expression for the CoF under wrap angle Θ = 90° becomes

(15) $$\mu =\frac{4}{\pi }\left(\frac{F_{1}-F_{2}-F_{b}}{F_{1}+F_{2}}\right)$$

A disadvantage of Equation (15) is that the assumption of uniform pressure is inconsistent with the constant bending moment required to maintain constant curvature of the sample in contact with the countersample. Therefore, recently, Nielsen et al. [38] derived an equation that takes into account the correction of the analytical solution in the form as follows:

(16) $$\mu =\frac{F_{1}-F_{2}-F_{b}}{{F_{1}+F}_{2}}$$

## 3. Results and Discussion

### 3.1. Stretch-Forming Forces

**Figure 11:**
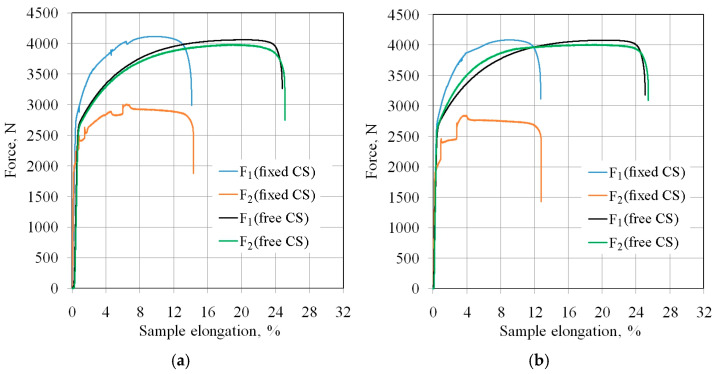

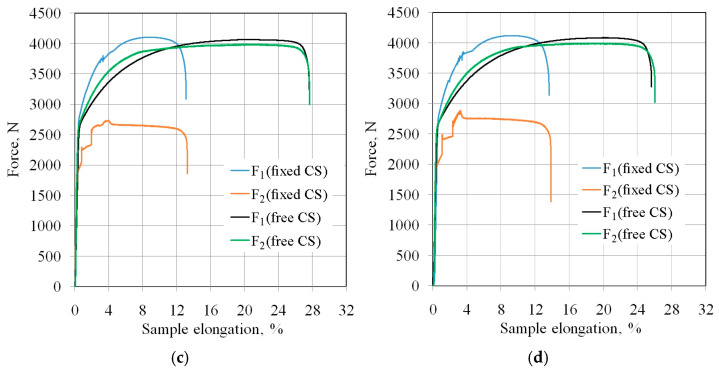
Values of force parameters in the friction process for the following countersamples: (**a**) electron beam melted countersample, (**b**) unmodified countersample, (**c**) Pb ion implanted and electron beam melted countersample, and (**d**) Pb ion implanted countersample.

Figure 11 shows the change in force parameters during two stages of the BUT test with the freely rotating countersample (CS) and stationary countersample. Under the freely rotating countersample condition, the total elongation of the sample was between 25.1% (EBM and unmodified countersamples) and 28% (IOPb+EBM). During the test with the stationary countersample, the elongation of the samples at breaking point was between 12.7% (Figure 11b) and 14.3% (Figure 11a). The largest friction-induced reduction in the sample elongation to breaking point was observed for the IOPb+EBM countersample, from 27.6% to 13.2% (Figure 11a). In contrast, the highest friction-induced reduction in the strip sample elongation was recorded for the IOPb+EBM countersample (Figure 11c), from approximately 27.5% to 13%. This indicates that the most unfavourable high-friction conditions occurred for the IOPb+EBM countersample. In the freely rotating countersample, the sample elongation was not affected by friction, and the curves were smooth with a continuous increase in force associated with the work-hardening phenomenon.

Frictional conditions caused an increase in front-tension force compared to the freely rotating countersample. This force also accounted for the resistance associated with bending and straightening the sheet metal around the cylindrical countersample. Under the same conditions, the back-tension force was significantly lower than that encountered with a rotating countersample. Initially, the surface asperities of the strip samples were flattened by sample deformation, followed by topographic evolution in the longitudinal and transverse directions. The deformation-induced change in the sample surface topography is associated with elastic-plastic strains that vary locally in the microstructure as a result of different grain sizes [51], grain orientations [52], and the non-uniform distribution of dislocations and point defects [22]. The increased strength of the sheet as a result of deformation-induced work hardening phenomena causes a decrease in the ductility of the sample material. Bending associated with plastic deformation leads to a change in the mechanical and tribological properties of the material through local deformation and wrinkling of the sheet, which was also observed by Krbata et al. [53].

According to the constant volume principle, elongation of the sample causes a reduction in its thickness and width. Despite the reduction in sample width, the front-tension force increases due to the work-hardening phenomenon. This phenomenon is limited to the formation of a neck in the sample, which occurs on the side where the front-tension force occurs (Figure 7).

### 3.2. Coefficient of Friction

**Figure 12:**
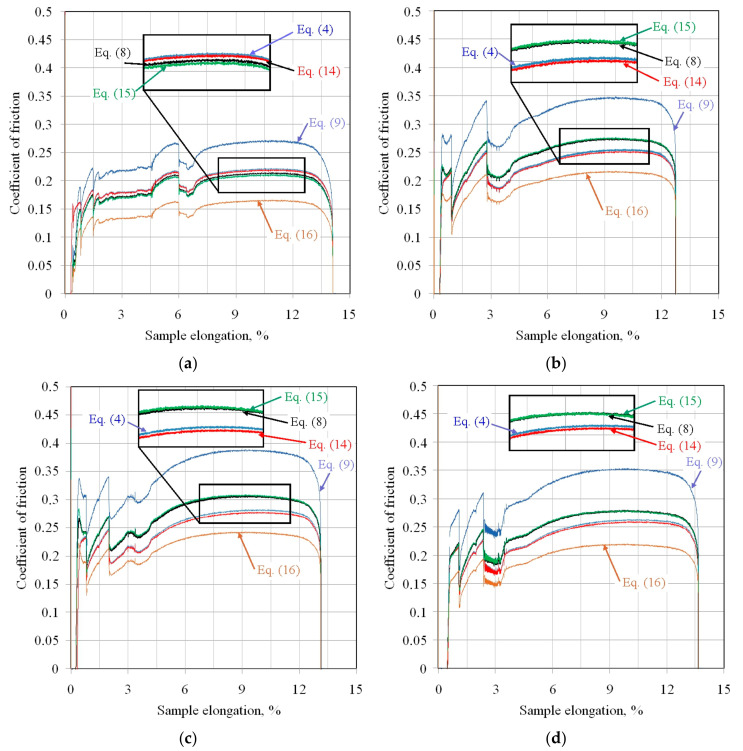
Evolution of the CoF under stretch-forming conditions for the following countersamples: (**a**) EBM, (**b**) unmodified, (**c**) IOPb+EBM, and (**d**) IOPb.

Figure 12 presents the variation in the coefficient of friction determined by six different analytical approaches. The considerations presented in this subsection concern the kinetic coefficient of friction. For all friction conditions, the highest friction coefficient values were obtained for the equation proposed by Han [50]. Similarly, the lowest CoF value for all friction pairs can be observed for the model proposed by Nielsen et al. [38].

For all friction pairs, Equations (8) and (15) and Equations (4) and (14) showed similar values. However, for friction pairs DC01/145Cr6 (Figure 12b), DC01/IOPb+EBM (Figure 12c), and DC01/IOPb (Figure 12d), the friction coefficients determined from Equations (8) and (15) showed higher values than the CoFs determined from Equations (4) and (14). Meanwhile, for the DC01/EBM friction pair (Figure 12a), this relationship was reversed. However, it should be noted, that, for this friction pair, the CoF values determined from Equations (4), (8), (14) and (15) were more similar compared to the other friction pairs.

Due to the continuous change in the sample surface topography and the contact pressure values during the BUT test, the friction coefficient values are constantly changing. However, a clear initial stage of increasing the friction coefficient, through its stabilisation, and then, finally decreasing, is visible (Figure 12). This is related to the change in the ratio between front-tension and back-tension forces during strip sample stretching.

**Figure 13:**
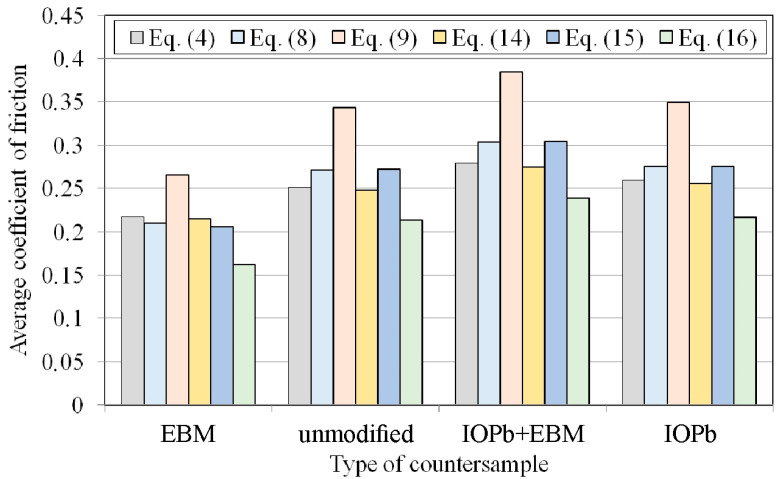
The influence of the type of countersample and the type of equation on the average CoF.

For comparison purposes, average friction coefficient values were determined for sample elongation between 7% and 12% (Figure 12). The difference between the highest and lowest average friction coefficient values determined by the considered analytical models was approximately 40% (Figure 13). The highest friction coefficient values for all analysed analytical equations were observed for the DC01/IOPb+EBM friction pair. The lowest COFs in the BUT test were provided by the DC01/EBM friction pair. It should be noted that a high value of the friction coefficient at the edge of the punch (Figure 2) facilitates the deep drawing process by increasing the value of the force necessary to break the drawpiece.

All of the analytical models which were analysed for the determination of the CoF involve certain simplifications and it is not possible to determine which model is most appropriate. The researchers should take into account the fact that quantitatively comparing friction coefficients determined by different models can lead to large differences.

The results presented in this manuscript refer to cold forming conditions. However, as the processing temperature increases, the friction coefficient increases [54]. Higher temperatures cause faster plasticisation of the sheet material and adhesion of the asperity peaks. At high temperatures, metals can undergo thermal softening, which increases their real contact area, leading to more severe wear mechanisms, such as ploughing and adhesion, and thus increasing the friction coefficient. Similarly, increasing the strain rate intensifies the mechanical interaction of the asperity peaks and their heating. Consequently, as the strain rate increases, the friction coefficient increases [55,56].

### 3.3. Surface Roughness of Strip Samples

Average roughness (Sa), kurtosis (Sku), and skewness (Ssk) were selected to analyse deformation-induced and friction-induced changes in sample surface roughness after BUT testing. The first of these parameters is a fundamental parameter for determining sheet topography in the sheet metal forming industry. Sedlaček et al. [57] mentioned a strong influence of the kurtosis Sku and skewness Ssk parameters on frictional contact. For lubricated sliding contact, Ssk and Sku were found to be the most important roughness parameters in terms of tribological behaviour.

**Figure 14:**
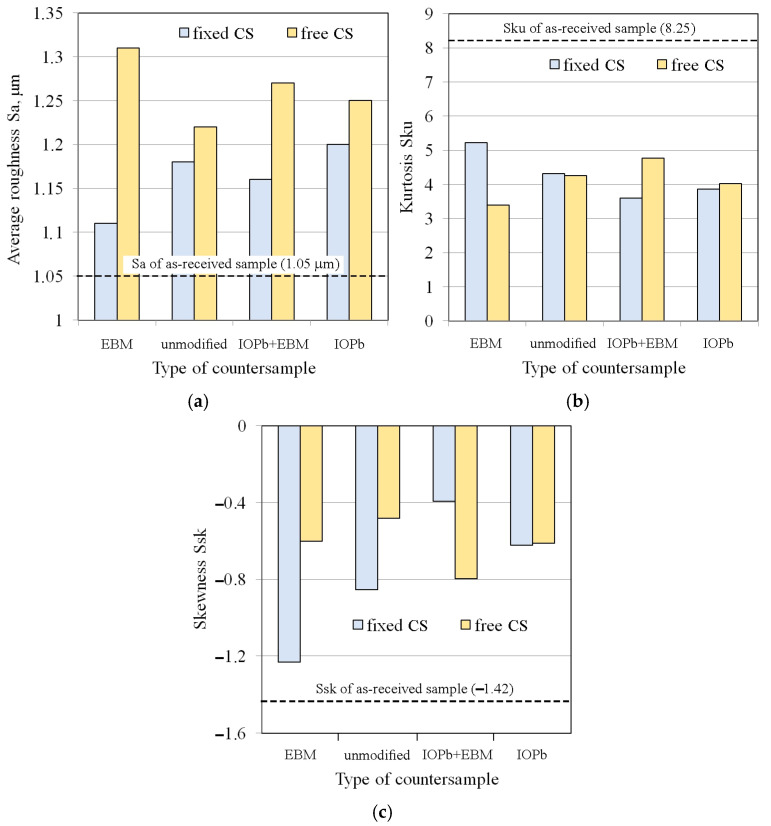
Effect of countersample condition on the surface roughness parameters of a strip sample: (**a**) average roughness Sa, (**b**) kurtosis Sku, and (**c**) skewness Ssk.

**Figure 15:**
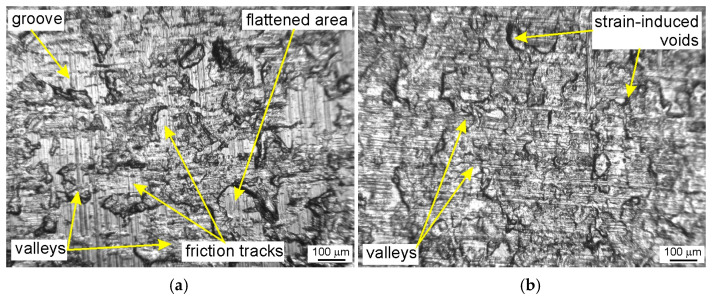

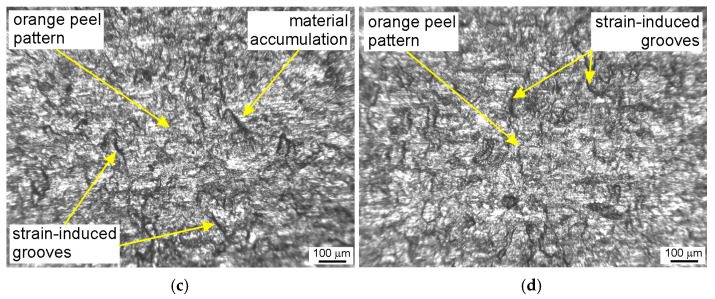
Optical images of the sheet metal surface after the friction process involving (**a**) stationary and (**b**) a freely rotating EBM countersample; the outer surface of the sample tested under conditions of (**c**) stationary and (**d**) a freely rotatable unmodified countersample.

The surface roughness of the samples after friction testing was examined on the side of the samples in contact with the countersamples. The friction process under stretch-forming conditions increased the average surface roughness of the strip samples (Figure 14a). During friction with the freely rotating countersample, the average roughness was higher than for the stationary countersample. Rolling contact limited the adhesion phenomenon, which limited the friction-induced flattening of the asperity summits. Consequently, the average roughness for the above-mentioned samples was higher than for friction with stationary countersamples (Figure 14a). In the stationary countersample conditions, the flattening phenomenon (Figure 15a) was accompanied by the adhesion of the asperity summits and resulted in more intense mechanical contact compared to the rolling contact conditions with the freely rotating countersample. The sample surface obtained after friction with a freely rotating countersample showed no friction traces (Figure 15b). Under friction testing with a stationary countersample, the largest increase in average roughness (by 19%) was found for the DC01/IOPb friction pair. The rotating countersample condition increased the average roughness by 5.7% and 14.3% for the DC01/IOPb and DC01/EBM friction pairs, respectively.

Kurtosis (Sku) is a surface roughness parameter describing the sharpness or roundness of the profile. Kurtosis values above 3 indicate surfaces with numerous, sharp peaks. Surfaces initially dominated by valleys were transformed into peak-dominated surfaces after deformation. The friction process caused a significant decrease in kurtosis values (Figure 14b). However, after the friction process, all countersamples showed Sku > 3, indicating that the spacing of the irregularities had decreased [58]. The main contributor to the increased kurtosis was strain-induced topography evolution, which caused an increase in the profile height. Meanwhile, friction causes a change in the height of surface asperities, and this is confirmed by the negative skewness values (Figure 14c). A negative Ssk value indicates a predominance of valleys in the surface roughness profile, as well as a tendency to create more bearing surface areas with increasing strain [58]. However, it should be noted that as a result of the friction process, the skewness value, although negative, increased the area ratio in the as-received condition (Ssk = −1.42). Surfaces with negative skewness exhibit favourable properties under lubricated conditions because the increased number of valleys provides spaces for lubricant to fill. Friction tests for the DC01/IOPb+EBM and DC01/IOPb friction pairs resulted in the greatest reduction in valleys during friction, involving both freely rotating and stationary countersamples. For high kurtosis values, the CoF decreases with decreasing external force. However, with increasing skewness, the CoF increases [59]. As previously mentioned, negative skewness indicates a predominance of valleys in the surface roughness profile, which favourably supports lubrication conditions. An increased number of valleys directly affects the amount of lubricant and ensures continuous lubricant delivery to the contact zone. The sheet metal in the DC01/EBM contact had the lowest skewness (Figure 14c), which resulted in the lowest friction coefficients (Figure 13). As a result of strain-induced and friction-induced changes, the kurtosis of the strip samples significantly decreased; however, it is above 3 (leptokurtic distribution). Reduced kurtosis indicates a reduced contact area, which is likely to be due to the formation of strain-induced grooves in the surface topography of the tensile samples. Friction-induced changes can be considered as the difference in kurtosis for the surfaces of the samples tested in fixed and freely rotating countersamples (Figure 14b). The direct correlation of kurtosis and CoF is difficult because of the continuous evolution of the topography.

The character of the deformation-induced surface roughness changes in sheet metal samples is correlated with the average grain size. Dai and Chiang [60] found that plastic deformation roughens a surface by introducing slip bands within grains and sliding between the grains. The rate of relative rotation between grains increases linearly with the amount of plastic deformation. The roughening of deformation-induced free surfaces was investigated by Guangnan et al. [61], who found that the major source of surface roughening is grain rotation, rather than growth and slip emergence. Another conclusion was that sample thickness does not affect the amount of roughness. Shimizu et al. [62] found that, with increasing strain, the strain difference between individual grains increases due to the anisotropic orientation of the microstructure. Baydogan et al. [63] developed a formula for determining the change in the average roughness of a sheet metal caused by deformation, which also takes into account the amount of plastic deformation.

The outer surface of the samples was not the subject of quantitative studies in this article. However, it is obvious that the free surface of the sheet becomes rough with increasing plastic strain. This phenomenon causes the formation of orange peel patterns (Figure 15c,d), which are defects of the sheet surface. High surface roughness, including the orange peel effect, affects the quality of a paint coating finish in the automotive industry [64]. High surface roughness caused by the forming process is also one of the limitations of single-point incremental sheet forming [65,66].

**Figure 16:**
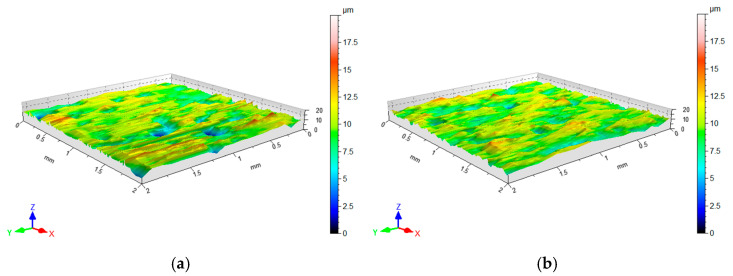
Surface topography of strip sample after the friction process under (**a**) stationary and (**b**) freely rotating EBM countersamples.

In addition to the orange peel defect, cold-rolled DC01 steel sheets subjected to a post-strain tensile state exhibit a directional topography resulting from grain deformation, which became oriented during the sheet metal production process. A pronounced directional topography of the sheet surface after friction was observed during friction with a stationary countersample (Figure 16a). This effect was not observed during BUT testing with a freely rotating countersample. In the contact zone area under stationary countersample conditions, the change in countersample width is limited by friction, hence the topography elongates more in the loading direction compared to the freely rotating countersample conditions (Figure 16b).

### 3.4. Contact Pressure

**Figure 17:**
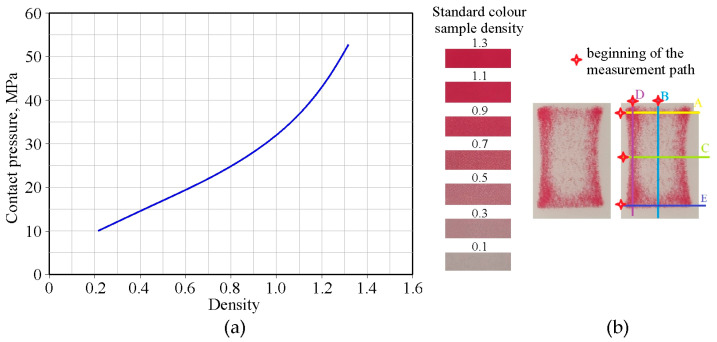
(**a**) Relationship between standard colour sample density and contact pressure and (**b**) contact pressure measurement cross-sections (A, C, E—transverse measurement paths; B, D—longitudinal measurement paths).

The contact pressure was evaluated using Prescale film during the BUT test with a freely rotating countersample for DC01/unmodified 145Cr6 countersample. Contact pressure measurements were performed along several longitudinal and transverse sections (Figure 17b) using a nomogram provided by the Prescale film manufacturer (Figure 17a).

**Figure 18:**
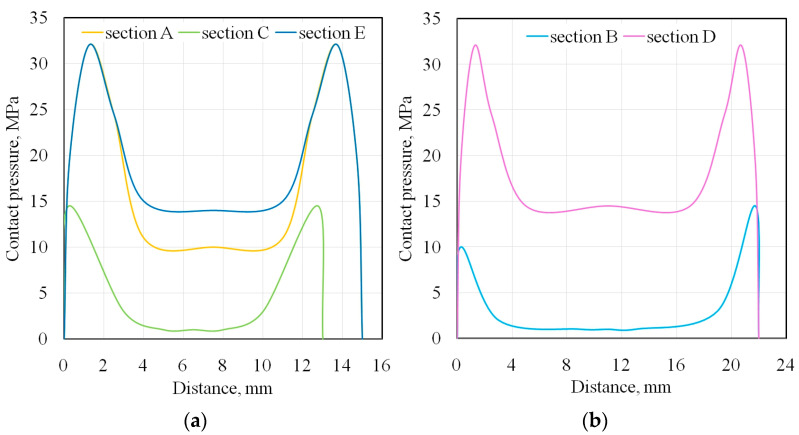
Contact pressure distribution on the (**a**) transversal cross-section and (**b**) longitudinal cross-section for the DC01/145Cr6 friction pair.

Two pressure peaks were observed in the longitudinal measurement section, appearing approximately 1.5 mm before the outlet and 1 mm after the inlet (Figure 18b). The results for the longitudinal section are consistent with those of Sniekers and Smiths [27], Pereira et al. [42], and Coubrough et al. [67], who also observed two pressure peaks at the sample’s entrance and exit from contact with the countersample. Similar peaks were observed in the cross-section at the edge of the sample (Figure 18a). The pressure distribution is highly inhomogeneous in both measurement directions. In the central contact zone, an area of reduced contact pressure can be observed (Figure 18a,b). This is related to the effect of bending the material around the countersample and the simultaneous stretching of the sample. The specific stress state occurring at the contact of the sheet metal with the cylindrical countersample causes local bending of the sheet metal. Therefore, the CoF determined in the BUT test should be considered as an average value for the entire contact area, rather than quantifying CoF values. The BUT test is suitable for assessing the effect of individual parameters on the coefficient of friction in comparative studies. Furthermore, as shown in Section 3.2, the value of the CoF in the BUT test depends on the analytical approach used.

Analytical models of the contact in BUT testing assume a constant contact pressure across the contact surface. Appendix A presents the relationship used in the BUT test to determine nominal contact pressure.

The difference between the highest and lowest average friction coefficient values, determined by the analytical models considered, was approximately 40%. Considering the types of counter-samples, two different relationships can be observed, indicating that the type of surface topography can influence the prediction of the CoF using a specific mathematical equation. In the case of the DC01/EBM friction pair (Figure 12a), Equations (4) and (14), as well as Equations (8) and (15), present almost identical friction coefficient curves. However, the remaining friction pairs, Equations (8) and (15), as well as Equations (4) and (14), represent similar friction coefficient curves. It is likely that the specific friction conditions of the DC01/EBM led to a disturbance in the ratio between front-tension and back-tension forces during the test. Equations (4), (8), (9), (14) and (15) were derived assuming uniform contact pressure between the strip sheet and the cylindrical countersample. Meanwhile, according to Figure 18b, there are two peaks before the outlet and after the inlet of the sheet, which was also previously observed in [27,42,67], among others. Given the simplified assumptions used to derive Equations (4), (8), (9), (14) and (15), the authors of Equation (16) proposed the replacement of the assumption of uniform pressure with two concentrated forces corresponding to the location of the two contact pressure peaks (Figure 18). Therefore, Equation (16), based on the current state of knowledge, is the most reliable and predicts the lowest CoF values among all the analysed conditions (Figure 12a–d).

Friction conditions in the FE numerical models influence the accuracy of representing the actual behaviour of the material during deformation. The work by Başpınar and Akkök [68] demonstrated the occurrence of multiple zones with different contact pressure distributions in the deep drawing process, and therefore different friction coefficient values. No universal method for determining the friction coefficient in sheet metal forming has been developed yet. Different tests, differing in the kinematics of the friction process, have been developed for each contact zone. Determining the friction coefficient and its evolution during the machining process is essential as a boundary condition in FE-based numerical models of sheet metal forming. Until now, it was commonly assumed that the friction coefficient was constant not only in selected areas of the drawpiece but also on all contact surfaces. Sabet et al. [5] developed a multi-factor friction model that took into account a variable contact pressure and sliding speed. The proposed friction model, implemented in the FE-based numerical model, demonstrated improved prediction of the final geometry and forming force compared to the conventional Coulomb friction model. The research results presented in this article, in addition to their cognitive aspect, highlight the significant evolution of the friction coefficient during the forming process in the area of rounded edges of stamping tools. In addition to the tool edges, significant strain-induced topographic changes occur in the area of the blankholder action, especially in the case of axisymmetric drawpieces, when high circumferential compressive stresses dominate the blankholder action zone. Friction conditions play a significant role in finite element method models, as they significantly influence the realistic representation of interactions between contacting surfaces.

## 4. Conclusions

This paper presents the results of laboratory-scale friction investigations into DC01 steel sheets under stretch-forming conditions. Various methods for fabricating countersamples from cold work 145Cr6 tool steel were considered. Various analytical models are available in the literature and were used to process the results of friction tests. The main conclusions that can be drawn from the experimental studies are as follows:

- The BUT test with a stationary countersample resulted in a reduction in sample elongation by over 50% compared to the tests with a freely rotating countersample.
- The smallest friction-induced reduction in strip sample elongation was recorded for the DC01/IOPb+EBM friction pair, demonstrating the high friction conditions in the BUT test.
- Frictional conditions caused an increase in front-tension force compared to freely rotating countersamples, relating to overcoming the frictional resistance.
- For all friction conditions, the highest friction coefficient values were obtained for Equation (9). The lowest CoF value for all friction pairs was obtained for Equation (16).
- Due to the continuous change in the sample surface topography and the contact pressure values during the BUT test, the CoF values are constantly changing.
- The average CoF values, determined for sample elongation between 7% and 12%, showed that the difference between the highest and lowest CoF values, based on the analytical models considered, was approximately 40%.
- Under friction testing with a stationary countersample, the largest increase in average roughness (by 19%) was found for the DC01/IOPb friction pair. The friction process caused a significant decrease in kurtosis values.
- Two pressure peaks were observed along the length of the contact zone, both longitudinally and transverse to the stretching direction. This is related to the effect of bending the material around the cylindrical countersample and the simultaneous stretching of the sample.

The surface roughness of the countersample is crucial for ensuring proper lubrication conditions. Excessive roughness of the countersample surface can enhance the mechanism of mechanical engagement between the sheet metal and tool surface asperities. However, a high roughness height with rounded peaks supports the lubrication process by providing large valleys for lubricant accumulation. In the context of the lubrication effectiveness, the EBM countersample, characterised by the lowest average surface roughness and the highest kurtosis value, provided the lowest CoF values. If a high CoF is required between the punch edge and the sheet metal, then the IOPb+EBM countersample is the preferred choice.

It should be noted that all analysed analytical models for determining CoF involve certain simplifications, primarily related to the uniformity of constant pressure over the contact zone and the equilibrium of forces stretching the strip sample. The results showed that the difference in CoF prediction based on different models can be as much as 40%. Researchers and practitioners should consider that quantitatively comparing COFs that were determined by different models can lead to large differences.

The results presented in this article do not exhaust the research topic of friction using the BUT test. Further studies are planned to determine the effect of countersample radius and sliding speed on the CoF and changes in sheet metal surface roughness. Expanding the research campaign will allow the development of a large number of training sets and the construction of a friction model using a machine learning method. This approach is intended to reduce the labour intensity and costs of research in industrial conditions. One area of interest could be the construction of countersample surface structures through mechanical processing and the application of thin-film anti-wear coatings to reduce the CoF.

## Figures and Tables

**Table 1:** The basic surface roughness parameters of the DC01 steel sheet.

Sa, μm	Sq, μm	Sv, μm	Sp, μm	Sz, μm	Sku, –	Ssk, –
1.05	1.45	10.6	3.98	14.6	8.25	−1.42

## Data Availability

The original contributions presented in this study are included in the article. Further inquiries can be directed to the corresponding author.

## Appendix A

Nielsen et al. [38] assumed a uniform pressure in the contact zone (Figure 10). Under such conditions the vertical force equilibrium is

(A1) $$\left(F_{1}+F_{2}\right)sin\frac{\theta }{2}=\int_{-\frac{\theta }{2}}^{\frac{\theta }{2}}pRwcos\varphi d\varphi$$

And, after calculating the integral, the vertical force equilibrium is

(A2) $$\left(F_{1}+F_{2}\right)sin\frac{\theta }{2}=2p∙R∙w∙sin\frac{\theta }{2}$$

where F_1_ and F_2_ are front-tension and back-tension forces, respectively; Θ is wrap angle; w is strip sample width; p is contact pressure; and R is the radius of countersample.

After simplification, the contact pressure p will be

(A3) $$p=\frac{F_{1}+F_{2}}{2w∙R}$$

## References

[1] Sevšek L., Šimic M., Herakovič N., Pepelnjak T. (2025). Development of an innovative hydraulic press for incremental forming: Machine and process evaluation using a hybrid two-step process. Mater. Des..

[2] Gantar G., Pepelnjak T., Kuzman K. (2002). Optimization of sheet metal forming processes by the use of numerical simulations. J. Mater. Process. Technol..

[3] Balyts’kyi O.I., Kolesnikov V.O., Kawiak P. (2005). Triboengineering properties of austenitic manganese steels and cast irons under the conditions of sliding friction. Mater. Sci..

[4] Balyts’kyi O.I., Kolesnikov V.O., Eliasz J. (2013). Study of the wear resistance of high-nitrogen steels under dry sliding friction. Mater. Sci..

[5] Sabet A.S., Domitner J., Öksüz K.I., Hodžić E., Torres H., Rodríguez Ripoll M., Sommitsch C. (2021). Tribological investigations on aluminum alloys at different contact conditions for simulation of deep drawing processes. J. Manuf. Process..

[6] Szwajka K., Trzepieciński T., Szewczyk M., Zielińska-Szwajka J., Barlak M. (2025). Investigating Resulting Surface Topography and Residual Stresses in Bending DC01 Sheet Under Tension Friction Test. Lubricants.

[7] Trzepieciński T., Szwajka K., Szewczyk M., Zielińska-Szwajka J., Slota J., Kaščák Ľ. (2025). The Effect of the Addition of Silicon Dioxide Particles on the Tribological Performance of Vegetable Oils in HCT600X+Z/145Cr46 Steel Contacts in the Deep-Drawing Process. Materials.

[8] Evin E., Tomáš M. (2022). Influence of Friction on the Formability of Fe-Zn-Coated IF Steels for Car Body Parts. Lubricants.

[9] Tomków J., Czupryński A., Fydrych D. (2020). The Abrasive Wear Resistance of Coatings Manufactured on High-Strength Low-Alloy (HSLA) Offshore Steel in Wet Welding Conditions. Coatings.

[10] Jonda E., Fydrych D., Łatka L., Myalska-Głowacka H. (2024). The Use of Cluster Analysis to Assess the Wear Resistance of Cermet Coatings Sprayed by High Velocity Oxy-Fuel on Magnesium Alloy Substrate. Adv. Sci. Technol. Res. J..

[11] Wilson W.R.D., Sheu S. (1988). Real area of contact and boundary friction in metal forming. Int. J. Mech. Sci..

[12] Więckowski W., Adamus J., Dyner M. (2020). Sheet metal forming using environmentally benign lubricant. Arch. Civ. Mech. Eng..

[13] Evin E., Daneshjo N., Mareš A., Tomáš M., Petrovčiková K. (2021). Experimental Assessment of Friction Coefficient in Deep Drawing and Its Verification by Numerical Simulation. Appl. Sci..

[14] Kohutiar M., Krbata M., Escherova J., Eckert M., Mikus P., Jus M., Polášek M., Janík R., Dubec A. (2024). The Influence of the Geometry of Movement during the Friction Process on the Change in the Tribological Properties of 30CrNiMo8 Steel in Contact with a G40 Steel Ball. Materials.

[15] Szewczyk M., Mezher M.T., Jaber T.A. (2025). The Use of Artificial Neural Networks to the Analysis of Lubricating Performance of Vegetable Oils for Plastic Working Applications. Adv. Mech. Mater. Eng..

[16] Adamus J., Więckowski W., Lacki P. (2023). Analysis of the Effectiveness of Technological Lubricants with the Addition of Boric Acid in Sheet Metal Forming. Materials.

[17] Tomáš M., Evin E., Kepič J., Hudák J. (2019). Physical Modelling and Numerical Simulation of the Deep Drawing Process of a Box-Shaped Product Focused on Material Limits Determination. Metals.

[18] Mulidrán P., Spišák E., Tomáš M., Slota J., Majerníková J. (2020). Numerical Prediction and Reduction of Hat-Shaped Part Springback Made of Dual-Phase AHSS Steel. Metals.

[19] Pereira J.F.A., Prates P.A., Butuc M.C., Vincze G. (2025). Numerical Study on Continuous-Bending-Under-Tension of 3rd Generation Steel. Metals.

[20] Martínez-Martínez A., Miguel V., Coello J. (2024). A numerical model to analyse the under-tension-bending-unbending processes. Special analysis for determining the spring-back of TRIP 690 steel sheet. J. Manuf. Process..

[21] Nie N., Su L., Deng G., Li H., Yu H., Tieu A.K. (2021). A review on plastic deformation induced surface/interface roughening of sheet metallic materials. J. Mater. Res. Technol..

[22] Wu Y., Recklin V., Groche P. (2021). Strain Induced Surface Change in Sheet Metal Forming: Numerical Prediction, Influence on Friction and Tool Wear. J. Manuf. Mater. Process..

[23] Wilson W.R.D., Malkani H.G., Saha P.K. (1991). Boundary friction measurements using a new sheet metal forming simulator. Trans. NAMRI/SME.

[24] Swift H.W. (1948). Plastic bending under tension. Engineering.

[25] Saha P.K., Wilson W.R.D. (1994). Influence of plastic strain on friction in sheet metal forming. Wear.

[26] Andreasen J.L., Olsson D.D., Chodnikiewicz K., Bay N. (2006). Bending under tension test with direct friction measurement. Proc. Inst. Mech. Eng. Part B J. Eng. Manuf..

[27] Sniekers R.J.J.M., Smits H.A.A. (1997). Experimental set-up and data processing of the radial strip-drawing friction test. J. Mater. Process. Technol..

[28] Shih H.-C. (2024). Experimental Study on Bendability of Advanced High Strength Steels.

[29] Sharma R., Poulin C.M., Knezevic M., Miles M.P., Fullwood D.T. (2021). Micromechanical origins of remarkable elongation-to-fracture in AHSS TRIP steels via continuous bending under tension. Mater. Sci. Eng. A.

[30] Hudgins A.S., Matlock D.K., Speer J.G., Fekete J.R., Walp M.S. The Susceptibility to Shear Fracture in Bending of Advanced High Strength. Proceedings of the Materials Science and Technology 2007.

[31] Wagner L., Wallner M., Larour P., Steineder K., Schneider R. (2021). Reduction of Young’s modulus for a wide range of steel sheet materials and its effect during springback simulation. IOP Conference Series: Materials Science and Engineering.

[32] Zheng X., Han L., Hongwei H., Liu Y., Li X., Wu X., Wan M. (2024). Influence of hardening model on draw-bending springback prediction of DP980 dual-phase steel. Mater. Proc..

[33] Matukhno N., Kljestan N., Vogel S.C., Knezevic M. (2022). Cyclic bending under tension of alloy AZ31 sheets: Influence on elongation-to-fracture and strength. Mater. Sci. Eng. A.

[34] Ha J., Piccininni A., Korkolis Y.P., Palumbo G., Knezevic M., Kinsey B.L., Daehn G., Cao J., Kinsey B., Tekkaya E., Vivek A., Yoshida Y. (2021). Formability Improvements of AA5754-H32 at Room Temperature via Continuous Bending Under Tension (CBT) and Pre-forming Heat Treatment. Forming the Future.

[35] Mayer S., Matukhno N., Kinsey B.L., Knezevic M., Ha J. (2024). Manipulation of strength and ductility of AA5182-O through cyclic bending under tension and annealing processing. J. Manuf. Process..

[36] Tamimi S., Sivaswamy G., Pirgazi H., Amirkhiz B.S., Moturu S., Siddiq M.A., Kockelmann W., Blackwell P. (2021). A new route for developing ultrafine-grained Al alloy strips using repetitive bending under tension. Mater. Des..

[37] Folle L., Schaeffer L. (2019). New proposal to calculate the friction in sheet metal forming through bending under tension test. Mater. Res..

[38] Nielsen C.V., Legarth B.N., Niordson C.F., Bay N. (2022). A correction to the analysis of bending under tension tests. Tribol. Int..

[39] Kim Y.S., Jain M.K., Metzger D.R. (2012). Determination of pressure-dependent friction coefficient from draw-bend test and its application to cup drawing. Int. J. Mach. Tools Manuf..

[40] Trzepiecinski T., Lemu H.G. (2020). Effect of Lubrication on Friction in Bending under Tension Test-Experimental and Numerical Approach. Metals.

[41] Lemu H.G., Trzepieciński T. (2013). Numerical and Experimental Study of Frictional Behavior in Bending Under Tension Test. Stroj. Vestn.—J. Mech. Eng..

[42] Pereira M.P., Yan W., Rolfe B.F. (2008). Contact pressure evolution and its relation to wear in sheet metal forming. Wear.

[43] Ceron E., Bay N. (2013). Determination of friction in sheet metal forming by means of simulative tribo-tests. Key Eng. Mater..

[44] (2020). Metallic Materials—Tensile Testing—Part 1: Method of Test at Room Temperature.

[45] (2012). Geometrical Product Specifications (GPS)—Surface Texture: Areal—Part 2: Terms, Definitions and Surface Texture Parameters.

[46] Romanowski W.P. (1990). Cold Sheet Metal Forming.

[47] Fujifilm Prescale. https://fujiprescalefilm.com/.

[48] Duncan J.L., Shabel B.S., Filho J.G. (1978). A Tensile Strip Test for Evaluating Friction in Sheet Metal Forming.

[49] Fox R.T., Maniatty A.M., Lee D. (1989). Determination of friction coefficient for sheet materials under stretch-forming conditions. Metall. Trans. A.

[50] Han S.S. (1997). Influence of tool geometry on friction behavior in sheet metal forming. J. Mater. Process. Technol..

[51] Cao S.Q., Zhang J.X., Wu J.S., Chen J.G. (2015). Effect of local texture on the orange peel defect in St14 steel sheet. Materials Science Forum.

[52] Schäfer R. (2008). Kontaktgebundene Oberflächenwandlung Polykristalliner Blechoberflächen.

[53] Krbata M., Ciger R., Kohutiar M., Sozańska M., Eckert M., Barenyi I., Kianicova M., Jus M., Beronská N., Mendala B. (2023). Effect of Supercritical Bending on the Mechanical & Tribological Properties of Inconel 625 Welded Using the Cold Metal Transfer Method on a 16Mo3 Steel Pipe. Materials.

[54] Filzek J., Keil D., Schröder H. Temperature Induced Friction Increase in Friction Test and Forming Demonstrator for Sheet Metal Forming. ESAFORM 2021 [Online], Online Since 08 April 2021. https://popups.uliege.be/esaform21/index.php?id=3732.

[55] de Oliveira Lopes A.P., de Almeida D.T., Johnson S.M., D’Oliveira A.S.C.M., Costa H.L., Scheuer C.J. (2025). Influence of sliding speed and contact pressure on the tribological performance of cold working tool steels in strip drawing tests. Wear.

[56] Yang X., Zhang Q., Zheng Y., Liu X., Politis D., El Fakir O., Wang L. (2021). Investigation of the friction coefficient evolution and lubricant breakdown behaviour of AA7075 aluminium alloy forming processes at elevated temperatures. Int. J. Extrem. Manuf..

[57] Sedlaček M., Vilhena L.M.S., Podgornik B., Vižintin J. (2011). Surface topography modelling for reduced friction. Stroj. Vestn.-J. Mech. Eng..

[58] Hu Z.M., Dean T.A. (2000). A study of surface topography, friction and lubricants in metalforming. Int. J. Mach. Tools Manuf..

[59] Tayebi N., Polycarpou A.A. (2004). Modeling the effect of skewness and kurtosis on the static friction coefficient of rough surfaces. Tribol. Int..

[60] Dai Y.Z., Chiang F.P. (1992). On the mechanism of plastic deformation induced surface roughness. J. Eng. Mater. Technol..

[61] Guangnan C., Huan S., Shiguang H., Baudelet B. (1990). Roughening of the free surfaces of metallic sheets during stretch forming. Mater. Sci. Eng. A.

[62] Shimizu I., Okuda T., Abe T., Tani H. (2001). Surface roughening and deformation of grains during uniaxial tension of polycrystalline iron. JSME Int. J. Ser. A Solid Mech. Mater. Eng..

[63] Baydogan M., Akoy M.A., Kayali E.S., Cimenoglu H. (2003). Deformation induced surface roughening of austenitic stainless steels. ISIJ Int..

[64] Love J.C., Smith G.F., Pharaoh M., Coates R. (2001). Orange peel: Who cares?. Proc. Inst. Mech. Engineers. Part D J. Automob. Eng..

[65] Kuczek Ł., Żaba K., Trzepieciński T., Balcerzak M., Novák V. (2025). Characterization of Hexagonal Close-Packed Zn-Cu-Ti Alloy Pyramid Drawpieces in Single-Point Incremental Sheet Forming Process. Materials.

[66] Kuczek Ł., Żaba K., Trzepieciński T., Wąsikowski M., Balcerzak M., Sitek R. (2025). Influence of Heat Treatment on Properties and Microstructure of EN AW-6082 Aluminium Alloy Drawpieces After Single-Point Incremental Sheet Forming. Appl. Sci..

[67] Coubrough G.J., Allinger M.J., Van Tyne C.J. (2002). Angle of contact between sheet and die during stretch-bend deformation as determined on the bending-under-tension friction test system. J. Mater. Process. Technol..

[68] Başpınar M., Akkök M. (2016). Modeling and Simulation of Friction in Deep Drawing. J. Tribol..

